# The protective effect of the Mediterranean diet on endothelial resistance to GLP-1 in type 2 diabetes: a preliminary report

**DOI:** 10.1186/s12933-014-0140-9

**Published:** 2014-11-19

**Authors:** Antonio Ceriello, Katherine Esposito, Lucia La Sala, Gemma Pujadas, Valeria De Nigris, Roberto Testa, Loredana Bucciarelli, Maurizio Rondinelli, Stefano Genovese

**Affiliations:** Institut d’ Investigación Biomédiques August Pi i Sunyer (IDIBAPS) and Centro de Investigación Biomedica en Red de Diabetes y Enfermedades Metabolicas Asociadas (CIBERDEM), Hospital Clinic, C/ Rosselló, 149-153, 08036 Barcelona, Spain; Division of Metabolic Diseases, Center of Excellence for Cardiovascular Diseases, 2ndUniversity of Naples SUN, Naples, Italy; Experimental Models in Clinical Pathology, INRCA-IRCCS National Institute, Ancona, Italy; Department of Cardiovascular and Metabolic Diseases, IRCCS Gruppo Multimedica, Sesto San Giovanni, MI Italy

**Keywords:** Diabetes mellitus, Acute hyperglycemia, GLP-1, Oxidative stress, Mediterranean diet

## Abstract

**Background:**

In type 2 diabetes, acute hyperglycemia worsens endothelial function and inflammation,while resistance to GLP-1 action occurs. All these phenomena seem to be related to the generation of oxidative stress. A Mediterranean diet, supplemented with olive oil, increases plasma antioxidant capacity, suggesting that its implementation can have a favorable effect on the aforementioned phenomena. In the present study, we test the hypothesis that a Mediterranean diet using olive oil can counteract the effects of acute hyperglycemia and can improve the resistance of the endothelium to GLP-1 action.

**Methods:**

Two groups of type 2 diabetic patients, each consisting of twelve subjects, participated in a randomized trial for three months, following a Mediterranean diet using olive oil or a control low-fat diet. Plasma antioxidant capacity, endothelial function, nitrotyrosine, 8-iso-PGF2a, IL-6 and ICAM-1 levels were evaluated at baseline and at the end of the study. The effect of GLP-1 during a hyperglycemic clamp, was also studied at baseline and at the end of the study.

**Results:**

Compared to the control diet, the Mediterranean diet increased plasma antioxidant capacity and improved basal endothelial function, nitrotyrosine, 8-iso-PGF2a, IL-6 and ICAM-1 levels. The Mediterranean diet also reduced the negative effects of acute hyperglycemia, induced by a hyperglycemic clamp, on endothelial function, nitrotyrosine, 8-iso-PGF2a, IL-6 and ICAM-1 levels. Furthermore, the Mediterranean diet improved the protective action of GLP-1 on endothelial function, nitrotyrosine, 8-iso-PGF2a, IL-6 and ICAM-1 levels, also increasing GLP-1-induced insulin secretion.

**Conclusions:**

These data suggest that the Mediterranean diet, using olive oil, prevents the acute hyperglycemia effect on endothelial function, inflammation and oxidative stress, and improves the action of GLP-1, which may have a favorable effect on the management of type 2 diabetes, particularly for the prevention of cardiovascular disease.

Cardiovascular disease is a major complication of type 2 diabetes and cause of death [1]. Hyperglycemia seems to be an important contributor toward cardiovascular complications of diabetes, and it has been suggested that it produces such damage through the generation of oxidative stress [2]. Particularly, there is evidence that an acute increase in glycemia can produce oxidative stress, leading to endothelial dysfunction and inflammation [2]. Both endothelial dysfunction and inflammation are well-recognized pathogenic factors for vascular disease, particularly in diabetes [2].

Until recently, any intervention with antioxidants aiming to prevent cardiovascular complications in both non-diabetic and diabetic people has yielded disappointing results [3]. The PREDIMED trial, however, showing that a Mediterranean diet (MedDiet) enriched in monounsaturated fatty acids or polyunsaturated fatty acids and polyphenols can prevent cardiovascular disease in both non-diabetic and diabetic people, can be considered the first proof that an “antioxidant” intervention can provide certain benefits [4]. This hypothesis is strongly supported by evidence in PREDIMED of a significant increase in the antioxidant capacity in the plasma of people receiving the MedDiet [5], and that this increase is particularly relevant when using olive oil [6].

Recently, a possible beneficial effect of glucagon-like peptide-1 (GLP-1) analogues in the management of diabetes has been suggested [7]. GLP-1 and its analogues, in addition to their insulin-tropic action in alleviating hyperglycemia, have beneficial effects in protecting from the progressive impairment of pancreatic β-cell function, preserving β-cell mass and suppressing glucagon secretion, gastric emptying and appetite, all of which are characteristics that could prove beneficial for the management of diabetes [7].

Apart from the well-documented incretin effect of GLP-1, its role in the cardiovascular system also arouses interest. GLP-1 effects on the cardiovascular system may include a direct action on the endothelium, where the presence of specific receptors for GLP-1 has been demonstrated [8]. Consistently, GLP-1 has demonstrated to improve endothelial function in diabetes [9,10], possibly increasing the antioxidant defenses of the endothelium [11] and decreasing oxidative stress generation [10]. However, it is worth mentioning that, in both type 1 and type 2 diabetes, hyperglycemia induces an endothelial resistance to the action of GLP-1, with oxidative stress serving as the mediator of this phenomenon [10,12,13].

The aim of this study is to test the following in patients with type 2 diabetes:

- whether a MedDiet can counterbalance the effects of acute hyperglycemia on the generation of oxidative stress, endothelial dysfunction and inflammation;
- and if it can also improve the effects of GLP-1 during acute hyperglycemia on endothelial dysfunction, inflammation and oxidative stress.

## Methods

### Subjects and diets

The study included 24 type 2 diabetic patients. Baseline characteristics of the study groups are shown in Table 1. The study was approved by the Ethics Committee, and informed written consent was obtained from the study subjects.

**Table 1:** **Baseline characteristics of type 2 diabetic patients and the effects of one month following a Mediterranean diet using olive oil, or following a control low-fat diet**

	**MedDiet baseline**	**MedDiet 3 months**	**Control diet baseline**	**Control diet 3 months**
Sex	9M 3F	9M 3F	8M 4F	8M 4F
BMI Kg/m2	29.8 ± 1.4	29.6 ± 1.3	29.2 ± 1.1	29.3 ± 1.1
HbA1c %	8.1 ± 0.5	8.0 ± 0.4	8.0 ± 0.4	8.0 ± 0.6
HbA1c mmol/mol	65 ± 3.2	65 ± 3.0	65 ± 3.0	65 ± 3.3
Resting diastolic blood pressure mm Hg	77.4 ± 1.1	78.6 ± 1.3	78.6 ± 1.2	77.9 ± 1.4
Resting systolic blood pressure mm Hg	116.2 ± 1.3	115.5 ± 1.2	117.1 ± 1.4	116.7 ± 1.5
Total cholesterol mmol/l	4.30 ± 0.3	4.30 ± 0.4	4.27 ± 0.4	4.28 ± 0.3
Triglycerides mmol/l	1.2 ± 0.4	1.3 ± 0.5	1.3 ± 0.2	1.2 ± 0.5
HDL-C mmol/l	1.4 ± 0.2	1.4 ± 0.4	1.3 ± 0.3	1.4 ± 0.4
LDL-C mmol/l	2.2 ± 0.2	2.3 ± 0.4	2.1 ± 0.4	2.2 ± 0.5
FMD %	5.6 ± 0.5	7.9 ± 0.4*	5.5 ± 0.3	5.6 ± 0.6
8-iso-PGF2a (pg/ml)	68.4 ± 3.5	41.3 ± 2.2*	69.4 ± 3.0	68.8 ± 4.1
Nitrotyrosine μmol/l	0.64 ± 0.03	0.35 ± 0.02*	0.65 ± 0.04	0.64 ± 0.06
ICAM-1a (ng/ml)	170.4 ± 11.5	110.5 ± 10.1*	172.4 ± 10.5	173.3 ± 12.4
IL-6 (pg/ml)	230.35 ± 9.4	170.20 ± 8.3*	228.30 ± 10.3	235.55 ± 10.2
FRAP (μmol/l)	903.2 ± 57.2	1,810.3 ± 45.4*	911.6 ± 55.5	913.6 ± 52.4
TRAP (μmol/l)	807.5 ± 87.4	1,508.2 ± 60.1*	810.3 ± 75.2	813.3 ± 77.8

Data are expressed as mean ± SE *p < 0.05 vs baseline.

All patients were taking metformin, which they continued during the study. None of the type 2 diabetic patients presented retinopathy, nephropathy, or neuropathy. Ten patients had hypertension treated with an ACE inhibitor, which was withheld on the study days. None of the subjects was on statin or antioxidant supplements, and they were requested to maintain their regular physical activity and lifestyle and to record in a diary any event that could affect the outcome of the study (e.g., stress, change in smoking habits, alcohol consumption, or intake of foods not included in the experimental design). None of the participants showed evidence of high alcohol consumption or was an active smoker. Furthermore, during the previous 6 months, participants could not have taken part in any weight-reduction program or other nutritional intervention.

At their first appointment with the dietitian, all participants were informed about the study, asked to keep a 3D food diary, and completed a basic questionnaire regarding age, socioeconomic status, medical history, family history, physical activity, smoking and alcohol consumption habits, which allowed identification of foods to be modified.

Participants were randomly assigned to two groups of twelve patients each, using a computer-generated random number sequence. Each group received, for a period of 12 weeks, either a Mediterranean diet (MedDiet) enriched in monounsaturated fatty acids (MUFAs) (50 mL, 4 tablespoons extra virgin olive oil/day; approximately 1L/week), or a control low-fat diet [4].

The general MedDiet guidelines that dietitians provided to participants included the following positive recommendations [4]: a) abundant use of olive oil for cooking and dressing dishes; b) consumption of ≥2 daily servings of vegetables (at least one of them raw, such as in a salad), not including side dishes; c) ≥2-3 daily servings of fresh fruits (including natural juices); d) ≥3 weekly servings of legumes; e) ≥3 weekly servings of fish or seafood (at least one them fatty fish); f) ≥1 weekly serving of nuts or seeds; g) select white meats (poultry without skin or rabbit) instead of red meats or processed meats (burgers, sausages); and h) cooking regularly (at least twice a week) with tomato, garlic and onion, and dressing vegetables, pasta, rice and other dishes with a sauce made by slowly simmering minced tomato, garlic and onion with abundant olive oil. Negative recommendations were also given to eliminate or limit the consumption of cream, butter, margarine, cold cut meat, pâté, duck, carbonated and/or sugary beverages, pastries, industrial bakery products (such as cakes, donuts, or cookies), industrial desserts (puddings, custard), French fries and/or potato chips, and out-of-home pre-cooked cakes and sweets. The aim of the control diet [4] was to reduce all types of fat, with particular emphasis on the consumption of lean meats, low-fat dairy products, cereals, potatoes, pasta, rice, fruits and vegetables. In the control diet, advice on vegetables, red meat and processed meats, high-fat dairy products, and sweets concurred with the recommendations of the Mediterranean diet, but the use of olive oil for cooking and dressing and the consumption of nuts, fatty meats, sausages, and fatty fish were discouraged.

Compliance by participants was monitored through weekly telephone conversations with the dietitian and a check-list of the foods they consumed daily.

### Study design

Before and at the end of the diet intervention, baseline glycemia, insulin, endothelial function (flow mediated dilatation: FMD), plasma nitrotyrosine and 8-iso prostaglandin F2alpha (8-iso-PGF2a), GLP-1 (active 7-36), interleukin-6 (IL-6), intercellular adhesion molecule-1 (ICAM-1), the ferric reducing antioxidant potential (FRAP) and the total radical-trapping antioxidant parameter (TRAP) plasma levels were measured in each subject.

Before and at the end of the diet intervention, each subject underwent, in a randomized order and on different days, two hyperglycemic clamps [14], with or without GLP-1.

Synthetic GLP-1 [7-36] amide was purchased from PolyPeptide Laboratories (Wolfenbuttel Germany), and the same lot number was used in all studies. GLP-1 was the rate of 0.4 pmol Kg^−1^ min^−1^, according to Nauck et al. [15]. During the hyperglycemic clamp, the level of glycemia was levelled at 15 mmol/l.

Subjects were admitted to the research centre the evening before the experiment. All subjects received an evening meal and received a continuous low-dose infusion of insulin to normalize plasma glucose. The insulin infusion was adjusted overnight to maintain blood glucose between 4.4 and 7.2 mmol/l and stopped 2 hours before the start of each experiment.

After a 12-hour overnight fast, subjects were placed in a supine comfortable position with a room temperature between 20° and 24°C. Intravenous lines were inserted into a large antecubital vein of 1 arm for infusions and into a dorsal vein of the contralateral arm for blood sampling. Patency was preserved with a slow saline infusion (0.9% NaCl). The study began after the subjects had rested for 30 minutes.

During each clamp, at baseline and at 1 and 2 hours, glycemia, insulin, FMD, plasma nitrotyrosine, 8-iso-PGF2a, GLP-1 (active 7-36), IL-6 and ICAM-1 plasma levels were measured.

#### Biochemical Measurements

Cholesterol and triglycerides were measured enzymatically (Roche Diagnostics, Basel, Switzerland). HDL-C was estimated after the precipitation of apolipoprotein B with phosphotungstate/magnesium [16]. LDL-C was calculated after lipoprotein separation [16]. Plasma glucose was measured by the glucose-oxidase method, HbA1c by HPLC, and insulin by microparticle enzyme immunoassay (Abbott Laboratories, Wiesbaden, Germany). Nitrotyrosine plasma concentration was assayed by enzyme-linked immunosorbent assay (ELISA), recently validated by our laboratory [16]. Plasma 8-iso-PGF2a (Cayman Chemical, Ann Arbor, Michigan, USA.), ICAM-1 (British Bio-technology, Abington, Oxon, UK) and IL-6 (R&D Systems, Minneapolis, MN, USA), were determined with commercially available kits. GLP-1 (active 7-36) was measured by a radioimmunoassay kit (Peninsula Laboratories, Belmont, CA, USA). FRAP was measured according to Benzie et al. [17] and TRAP according to Ghiselli et al. [18].

Endothelial function was evaluated measuring the FMD of the brachial artery [19]. At the end of each test, the subjects rested quietly for 15 min. Then, sublingual nitroglycerin (0.3 mg) was administered, and 3 min later, the last measurements were performed. Response to nitroglycerin was used as a measure of endothelium-independent vasodilation.

### Statistical analysis

Data are expressed as Mean ± SE. The sample size was selected according to previous studies [9,10,20,21]. The Kolmogorov– Smirnov test did not show any significant departure from normality in the distribution of variance values. Comparisons of baseline data among the groups were performed using an unpaired Student’s *t*-test. The changes in variables during the tests were assessed by two-way ANOVA with repeated measurements. If differences reached statistical significance, post hoc analyses with two-tailed paired *t* test, using Bonferroni’s correction for multiple comparisons, were used to assess differences at individual time periods during the study. Statistical significance was defined as p < 0.05. All analyses were conducted using SPSS version 9.0 (SPSS Inc, Chicago, IL, USA).

## Results

With the MedDiet, FRAP, TRAP and FMD significantly increased, while nitrotyrosine, 8-iso-PGF2a, ICAM-1 and IL-6 significantly decreased (Table 1). There was no change with the control diet (Table 1).

**Figure 1:**
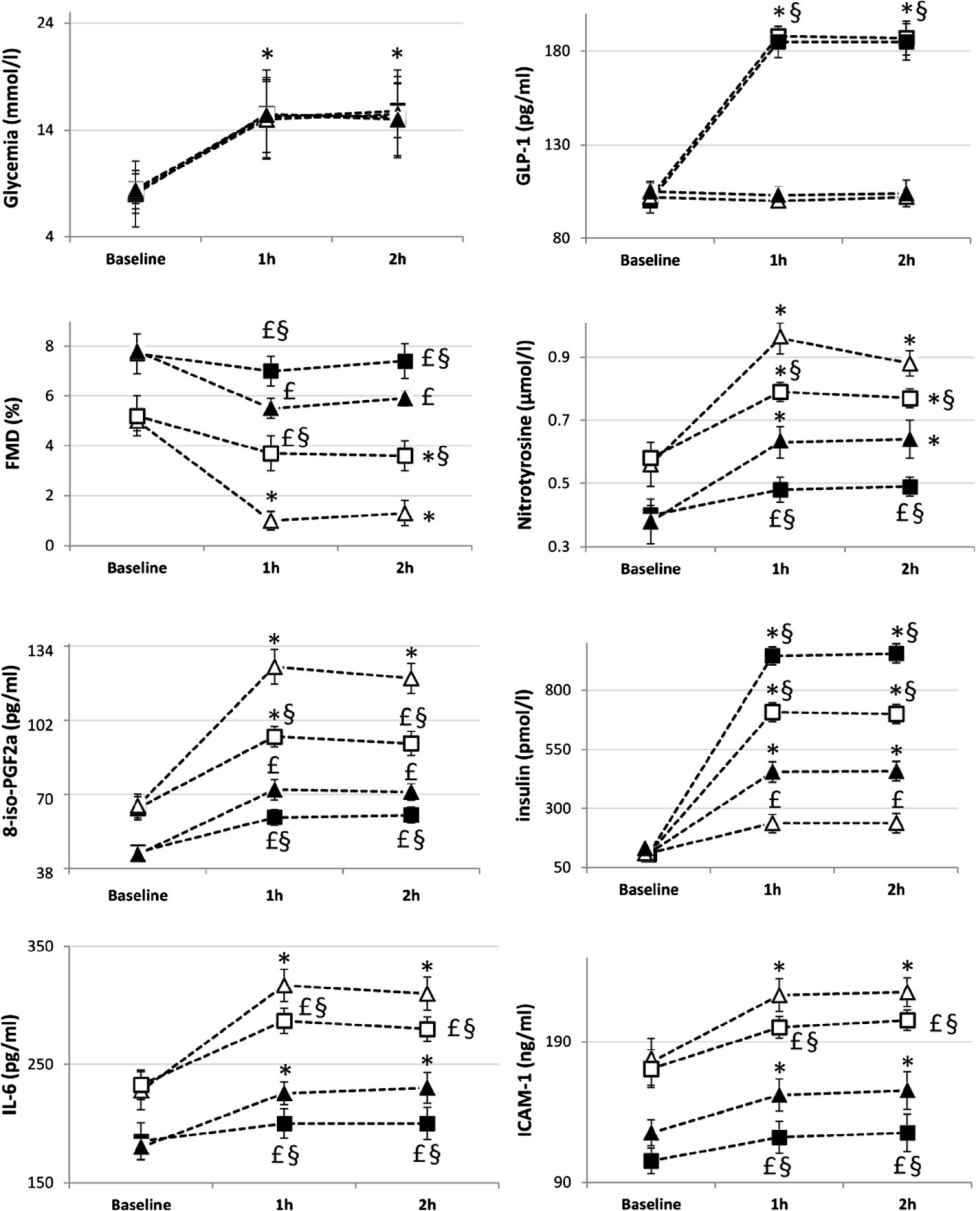
**Glycemia, GLP-1, FMD, nitrotyrosine, 8-iso-PGF2a, insulin, IL-6 and ICAM-1 changes during: baseline hyperglycemic clamp (white triangle); Baseline hyperglycemic clamp + GLP-1 (white square); Hyperglycemic clamp after MedDiet intervention (black triangle); Hyperglycemic clamp + GLP-1 after MedDiet intervention (black square).** Data are mean ± SE. £ p < 0.05 vs basal. *p < 0.01 vs basal. §p < 0.01 vs hyperglycemic clamp.

**Figure 2:**
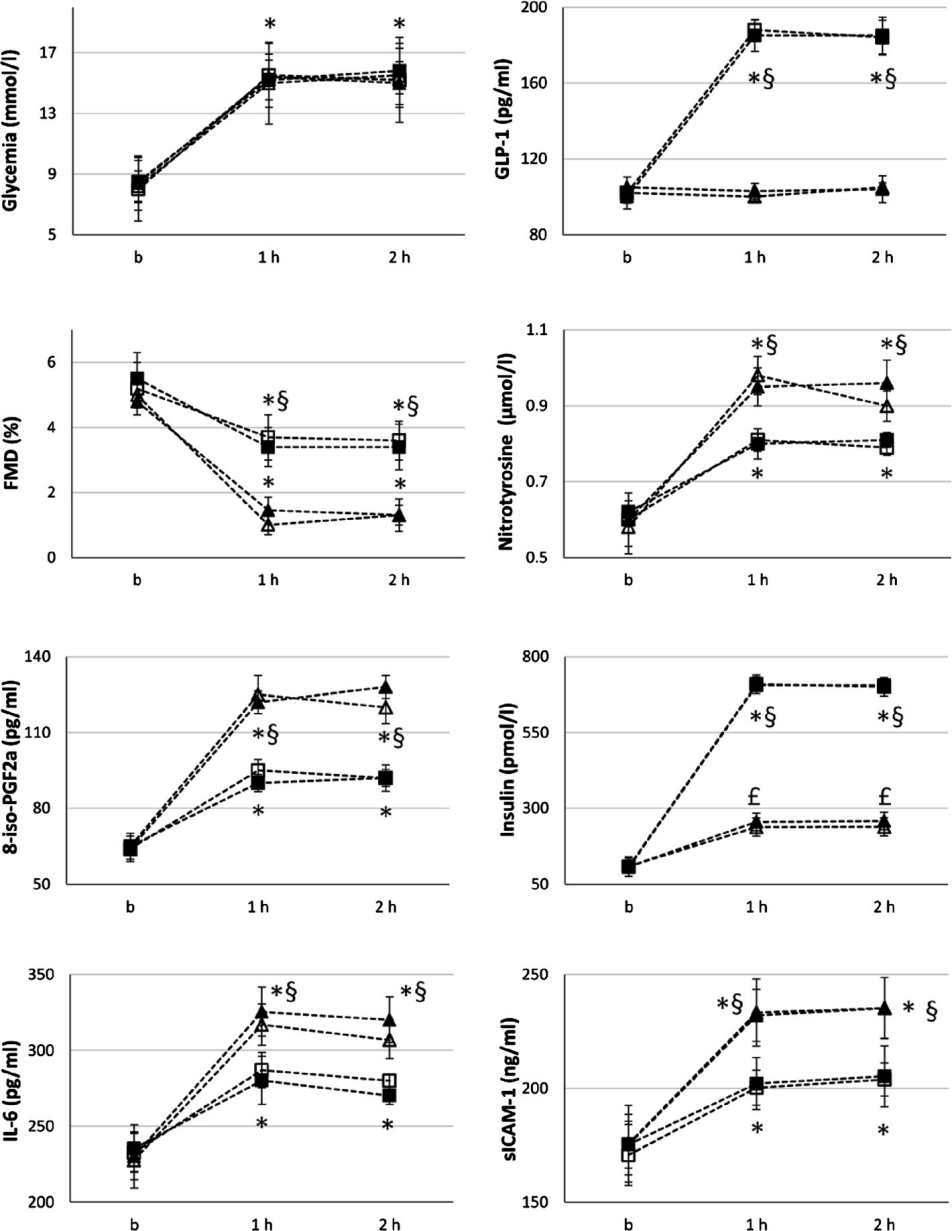
**Glycemia, GLP-1, FMD, nitrotyrosine, 8-iso-PGF2a, insulin, IL-6 and ICAM-1 changes during: baseline hyperglycemic clamp (white triangle); Baseline hyperglycemic clamp + GLP-1 (white square); Hyperglycemic clamp after Control diet intervention (black triangle); Hyperglycemic clamp + GLP-1 after Control diet intervention (black square).** Data are mean ± SE. £ p < 0.05 vs basal. *p < 0.01 vs basal. §p < 0.01 vs hyperglycemic clamp.

At baseline, in both groups, during the clamps without GLP-1, the concentration of this hormone remained unchanged, while its concentration was constantly high when infused (Figures 1 and 2). Insulin concentration increased in both groups during the hyperglycemic clamp, and its increase was significantly higher during GLP-1 infusion (Figures 1 and 2). During both clamps, with or without GLP-1, an increase in nitrotyrosine, 8-iso-PGF2a, ICAM-1 and IL-6, and a decrease in FMD were observed at 1 h and 2 h (Figures 1 and 2). However, at both 1 and 2 h, the values of nitrotyrosine, 8-iso-PGF2a, ICAM-1 and IL-6 significantly increased, while the values of FMD significantly decreased in the clamp without GLP-1, as compared to the values observed during the clamp with GLP-1 (Figures 1 and 2).

**Figure 3:**
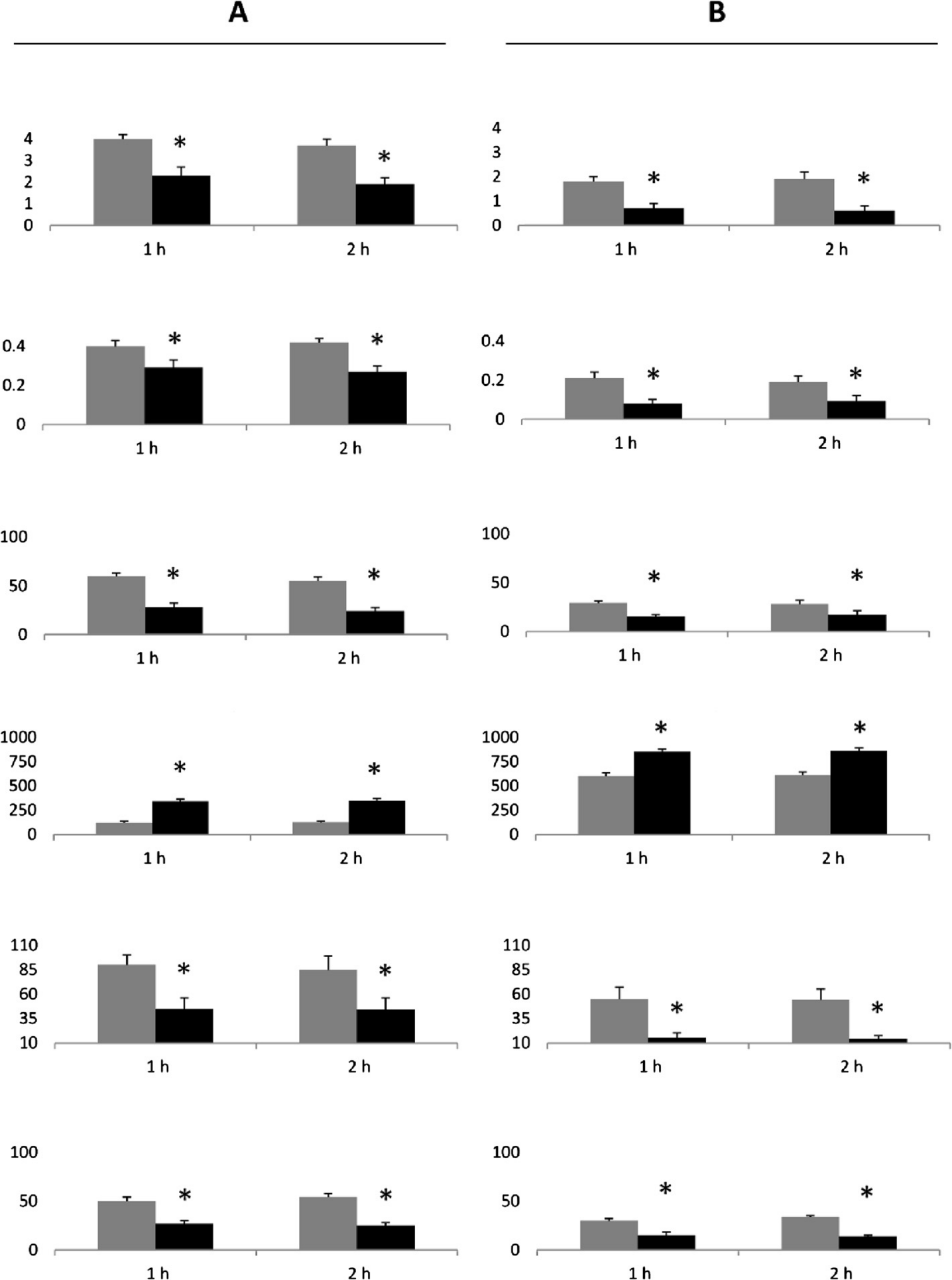
**Δ changes between baseline and after diet intervention. A**: Comparison between the Δ of the changes in FMD, nitrotyrosine, 8-iso-PGF2a, insulin, IL-6 and ICAM- during baseline hyperglycemic clamp (grey column) and hyperglycemic clamp after MedDiet intervention (black column). **B**: Comparison between the Δ of the changes in FMD, nitrotyrosine, 8-iso-PGF2a, insulin, IL-6 and ICAM- during baseline hyperglycemic clamp + GLP-1 (grey column) and hyperglycemic clamp + GLP-1 after MedDiet intervention (black column). *p < 0.01.

Following the MedDiet intervention, similarly to the baseline, at both 1 h and 2 h, the values of nitrotyrosine, 8-iso-PGF2a, ICAM-1 and IL-6 significantly increased, while the values of FMD significantly decreased in the clamp without GLP-1, as compared to the values observed during the clamp with GLP-1 (Figure 1). However, the same values of glycemia were less effective in producing oxidative stress and endothelial dysfunction after 1 month of the MedDiet. Since the basal values before and after the MedDiet were significantly different, the Δ between the basal value and the value at 1 h and 2 h during each clamp, with or without GLP-1, were compared to that in the previous clamp (Figure 3). Of particular interest, hyperglycemia was less effective in worsening oxidative stress, FMD and inflammation after the MedDiet compared to the previous clamp (Figure 3). At the same time, after the MedDiet, GLP-1 infusion was more effective in reducing oxidative stress and inflammation and in protecting FMD from the acute effects of hyperglycemia. Furthermore, after the MedDiet, GLP-1 infusion was accompanied by a significant increase in insulin secretion at both 1 h and 2 h (Figure 3).

There was no difference between the results of the clamps at baseline and after the control diet (Figure 2).

No difference was found in endothelium-independent vasodilatation in all the studies.

## Discussion

This study shows that the MedDiet using olive oil can improve endothelial dysfunction, inflammation and oxidative stress in type 2 diabetes. While several papers are available on the effects of the MedDiet on these parameters in metabolic syndrome [22,23], in hypercholesterolemia [24] or in healthy subjects [25], it is quite surprising that, until now, only one paper has reported on the effects of the MedDiet on endothelial dysfunction in type 2 diabetes [21]. Similarly, only one study, including only a small number of type 2 diabetic patients, has reported on the beneficial effects of the MetDiet on inflammation [26]. These papers, however, only aimed to show the potential benefits of the MedDiet on diabetes [21,26]. However, acute hyperglycemia alone can directly produce damaging effects such as endothelial dysfunction and inflammation, and is considered an important independent contributor toward the development of diabetic complications, particularly cardiovascular complications [2-27]. Several studies confirm that acute hyperglycemia works by generating oxidative stress [2,10,28-30]. Our study, for the first time, shows that the MedDiet, using olive oil, can counteract the effects of acute hyperglycemia. The effects of the MedDiet are convincingly related to its capacity for increasing antioxidant defenses. As already reported in a previous larger study [5], this diet increases both the plasma FRAP and TRAP in subjects. This effect could account for the reduced generation of oxidative stress observed during the hyperglycemic clamp and, therefore, for the reduced impact of acute hyperglycemia on endothelial function and inflammation.

Also of relevance, in our opinion, is the effect of the MedDiet on GLP-1 action.

It is now well recognized that GLP-1 activity is partially reduced in poorly controlled diabetic patients. This has been reported for insulin secretion and endothelial function [10,31]. The action of GLP-1 can be restored by improving glycemic control [10,31]. Two mechanisms have been suggested to explain this resistance to GLP-1 action in diabetes: the activation of PKCß, induced by hyperglycemia, able to reduce the expression of GLP-1 receptors [32]; and the generation of oxidative stress by hyperglycemia [10]. Nevertheless, the two proposed mechanisms -PKCß activation leading to the reduction of the expression of GLP-1 receptors, and oxidative stress generation- could be convincingly correlated, as it is well known that PKCß is activated by the free radicals [33]. Therefore, it has been suggested that hyperglycemia induces such a GLP-1 resistance, mainly through the generation of an oxidative stress [10]. This hypothesis has been confirmed in vivo showing that the GLP-1 action can be improved by an antioxidant, vitamin C [12,13]. The results of the present study, in our opinion, not only confirm this finding, but may also have a significant clinical impact. While the chronic use of vitamin C may not be a definitive solution [34], the evidence that the MedDiet improves GLP-1 action on both insulin secretion and endothelial dysfunction in diabetes might shed new light on the daily management of this disease.

Our study has several limitations. The number of subjects was quite small (2 groups of 12 patients each) and their inclusion was very selective, requiring that participants be free of complications and not taking statins, and that they be non-smokers and non-drinkers. This limits the extrapolation of our research to the general population, which is obviously much more heterogeneous.

Genetic influences on our data cannot be excluded. The association of the FTO-rs9939609 and MC4R-rs17782313 polymorphisms with type 2 diabetes depends on diet, and a high adherence to the MedDiet is able to counteract a genetic predisposition to cardiovascular disease [35]. Moreover, the MedDiet, particularly when using virgin olive oil, can exert certain health benefits via changes in the transcriptomic response of certain genes related to cardiovascular risk [36].

In conclusion, this study confirms that a MedDiet using olive oil improves endothelial dysfunction and inflammation, concomitantly increasing antioxidant defenses and decreasing oxidative stress. However, for the first time, this study shows that a MedDiet can counterbalance the negative effects of acute hyperglycemia on endothelial function, inflammation and oxidative stress, and can recover the protective action of GLP-1, not only on insulin secretion, but, more interestingly, also on endothelial function and inflammation in type 2 diabetes. Considering that both acute hyperglycemia [2] and a reduced protective effect of GLP-1 [37] can impact the development of cardiovascular complications, these results can be considered very relevant for the clinical management of diabetes. Future studies are needed to confirm this hypothesis.

## Abbreviations

MedDiet: Mediterranean diet
GLP-1: Glucagon like peptide-1
MUFAs: Monounsaturated fatty acids
FMD: Endothelial flow mediated dilatation
8-iso-PGF2a: 8-iso prostaglandin F2alpha
IL-6: Interleukin-6
ICAM-1: Intercellular adhesion molecule-1
FRAP: Ferric-reducing antioxidant potential
TRAP: Total radical-trapping antioxidant parameter
